# Spartan: surgical peg-and-ring triplet and workflow anticipation benchmark

**DOI:** 10.1007/s11548-026-03688-y

**Published:** 2026-05-06

**Authors:** Federico Cunico, Michele Sandrini, Nicola Piccinelli, Riccardo Muradore

**Affiliations:** Department of Engineering for Innovation Medicine, University of Verona, Strada Le Grazie, 15, Verona, 37134 Italy

**Keywords:** Surgical workflow anticipation, Surgical action triplet recognition, Surgical workflow analysis

## Abstract

**Purpose:**

Automation in robot-assisted surgery (RAS) requires not only accurate scene understanding but also real-time reasoning and action within dynamic surgical workflows. This work introduces SPARTAN: the *S*urgical *P*eg-*A*nd-*R*ing *T*riplet and Workflow *AN*ticipation Benchmark, alongside a unified baseline for real-time surgical workflow analysis, for the first time jointly addressing surgical phase recognition, phase anticipation, and action triplet recognition. This integrated design bridges high-level workflow understanding with fine-grained, robot-action-level perception.

**Methods:**

The SPARTAN benchmark is based on a modified Peg-and-Ring training task performed on the da Vinci Research Kit (dVRK), providing frame-level annotations of surgical phases and dual-arm action triplets that delineate initial, intermediate, and final workflow states.

**Results:**

We demonstrate that our baseline achieves performance comparable to state-of-the-art methods across all three SPARTAN tasks while operating in real time. The benchmark offers complexity comparable to related datasets in terms of phase structure, number of videos, and triplet diversity, yet remains reproducible and directly applicable to physical robotic systems.

**Conclusion:**

SPARTAN provides a practical foundation for developing and evaluating real-time perception and reasoning models in RAS.

**Supplementary Information:**

The online version contains supplementary material available at 10.1007/s11548-026-03688-y.

## Introduction

Robotic-assisted surgery (RAS) has significantly advanced minimally invasive interventions by enhancing dexterity, visualization, and motion precision, ultimately improving surgical outcomes [1]. The next frontier of RAS is autonomy, where robots move beyond teleoperation to active collaboration or independent task execution [2–5]. Achieving this goal requires not only precise perception but also real-time understanding and reasoning within dynamic and uncertain surgical workflows. Autonomous systems must interpret the surgical scene, anticipate upcoming actions, and decide when to act, all under strict latency constraints to ensure safety.

Surgical workflow understanding has traditionally relied on *surgical phase recognition*, which classifies video frames into coarse procedural stages such as “dissection" or “closure". Recent deep learning models leverage temporal convolutions or transformer-based architectures to capture multi-scale temporal dependencies [6–8], achieving high accuracy on benchmarks such as Cholec80 [1]. However, phase labels provide only a descriptive overview for human observers and lack the fine-grained semantics necessary for autonomous robotic reasoning. To represent surgical actions at a finer scale, *action triplet recognition* formalizes each activity as a combination of ⟨instrument, verb, target⟩ [9]. Recent advancements [10–13] have improved triplet association and modeling, improving the performance year after year. Yet, many methods primarily operate offline, focusing on static recognition rather than real-time, anticipatory understanding needed for control and planning.

When considering autonomous systems’ capability for context-awareness, the ability to foresee future surgical events is a cornerstone. This task is known as *surgical phase anticipation*. Early approaches modeled instrument interactions and workflow transitions [14], while generative models such as SUPR-GAN [15] and SWAG [16] introduced predictive reasoning to forecast upcoming surgical phases. These works demonstrate the value of anticipating workflow evolution for safety and efficiency, yet they remain computationally intensive, rely on external annotations (e.g., the instruments), and focus on individual prediction tasks rather than integrated, actionable understanding.

Bridging these hierarchies of perception, phase recognition, triplet-level action understanding, and temporal anticipation requires a unified, efficient framework that operates in real time. Moreover, the lack of reproducible, robot-executable benchmarks limits progress: most existing datasets, such as Cholec80 and CholecT50, derive from clinical recordings that are not easily transferable to physical robotic systems [1, 9]. To address these limitations, our main contributions are:

- The introduction of SPARTAN, the *S*urgical *P*eg-*A*nd-*R*ing *T*riplet and Workflow *AN*ticipation benchmark. A reproducible, action-oriented dataset bridging high-level surgical workflow and robotic-level interaction semantics;
- We introduce a lightweight, unified baseline that jointly handles phase recognition, anticipation, and triplet recognition. To our knowledge, this is the first framework to demonstrate the feasibility of simultaneously addressing all three tasks in real time.

Unlike CholecT50, which supports observational analysis on pre-recorded videos, SPARTAN is indended as an *executable* benchmark. It allows the research community to reproduce the physical setup on any dVRK system, enabling the validation of closed-loop control policies, something impossible with video-only clinical datasets. Furthermore, SPARTAN is designed with high “transition density,” strictly penalizing latency in phase recognition, which is critical for safety in autonomous execution, and aims to serve as a standardized calibration test for surgical perception models, complementary to clinical datasets, enabling rigorous cross-lab reproducibility that is often impossible with private patient data.

The paper is structured as follows: Sect. Related work presents the related literature of RAS, from phase recognition to anticipation and action triplet recognition. Section The SPARTAN benchmark presents the SPARTAN benchmark, Sect. Method details the unified architecture we propose, and Sect. Experiments presents experiments and discusses the results. Finally, Sect. Conclusions presents the conclusions of our work and future directions.

## Related work

Understanding surgical workflows at different semantic and temporal scales is fundamental for advancing automation in RAS. The literature can be categorized into three primary tasks: *Surgical Phase Recognition*, *Surgical Phase Anticipation*, and *Surgical Action Triplet Detection*. Each has produced strong but isolated solutions, which motivates unified approaches such as the one we propose.

Surgical phase recognition provides high-level context about procedural progress. In the deep learning era, the first successful convolutional network-based model was EndoNet [1]. Later, by improving online recognition, works such as TeCNO [6] achieved robust causal reasoning via dilated convolutions. Transformer-based architectures now dominate, particularly MuST [7], which captures long-term dependencies across multiple scales, and SKiT [8], which performs efficient key information pooling, achieving high accuracy and real-time inference. Other works explored hybrid CNN-Transformer schemes [17], but at the cost of higher computational load.

Phase anticipation extends recognition by predicting the timing and type of upcoming procedural events, supporting proactive robotic or cognitive assistance [3]. Early anticipation models used RNNs [18], requiring access to full sequences and thus unsuitable for online deployment. Recent methods shifted toward causal, predictive frameworks. SUPR-GAN [15] introduced a generative adversarial network for temporal prediction of phase trajectories, while IIA-Net [14] leveraged instrument interactions and causal dilated convolutions for long-horizon anticipation. The SWAG model [16] unified phase recognition and anticipation within a generative autoregressive framework, enabling minute-scale forecasts. Bayesian and uncertainty-aware formulations [19] incorporated probabilistic reasoning for reliable, risk-aware prediction. Despite these advances, most frameworks remain computationally heavy and non-causal, precluding integration into low-latency robotic perception loops.

At finer granularity, triplet detection identifies ⟨instrument, verb, target⟩ interactions to describe operative intent. Rendezvous [9] formalized this task, which used multi-head attention to link components, and Rendezvous-in-Time (RiT) [10] for temporal reasoning. TDN [11] introduced a triplet disentanglement strategy to mitigate imbalanced supervision, while DiffTriplet [12] applied diffusion-based generative modeling to refine triplet association. Knowledge-distillation approaches such as MT4MTL-KD [13] further improved generalization and robustness. These models achieve state-of-the-art accuracy on CholecT45 and CholecT50 benchmarks, yet their reliance on heavy frame-wise detection heads often incur computational costs that hinder high-frame-rate online execution. Triplet recognition also suffers from context blindness, as models often ignore phase-level constraints that could simplify prediction [9]. Integrating contextual workflow awareness remains an open research direction.

Recent research has begun to extend surgical automation beyond perception and assistance, targeting partial or full task autonomy. In these regards, SRT-H [20] demonstrated the first fully autonomous execution of a realistic Cholec. procedure on ex vivo pig tissue. Despite its remarkable success, several key challenges remain for autonomous surgical robotics. First, the proposed solution addresses only a specific surgical phase, lacking the global workflow understanding required for full autonomy. Second, it provides no guarantees on bounded inference latency or real-time performance. Demonstrations are shown at accelerated playback speeds (up to 14×), indicating sub-real-time execution. Finally, the end-to-end imitation learning approach used in SRT-H, while effective for task reproduction, does not explicitly model procedural reasoning or temporal structure, leaving phase-transition decisions implicit and opaque to supervisory control.

We believe the proposed SPARTAN benchmark complements this vision by offering a reproducible, robot-testable environment on the dVRK platform with dense temporal annotations, thus enabling the community to systematically study and validate closed-loop perception under real-time constraints. For comparison with existing datasets, Table 1 presents related work, including datasets for workflow analysis with phase and action triplet annotations.

**Table 1:** Comparison of surgical video datasets featuring workflow annotations and/or action triplet annotations

Dataset	# Videos	# Phases	# Triplet	Robotic?	Operation type	Public?
*M2CAI16-workflow*	41	8	—	N	Lap. Cholec	Yes
*Cholec80* [1]	80	7	—	N	Lap. Cholec	Yes
*HeiChole* [21]	33	7	—	N	Lap. Cholec	Yes
*AutoLaparo* [22]	21	7	—	N	Lap. Hyster	Yes
*CholecT50* [9]	50	—	100	N	Lap. Cholec	Yes*
*SPARTAN*	45	6	58	Y	Peg-And-Ring	Yes

Datasets marked private are not publicly accessible (*The test set of CholecT50 is not publicly released)

## The SPARTAN benchmark

In this section, we describe the SPARTAN benchmark. We designed a modified version of the classical peg-and-ring training exercise, introducing several phases to complete the task. The peg-and-ring setup is created as a flat board with eight pegs, four of which are colored with different colors (yellow, blue, green, red). Other pegs are black. Then, four rubber rings are present, all colored the same as the colored pegs, resulting in one ring per color. As for order, considering the pegs disposed in two rows of four pegs: yellow, black, blue, black in the first row; black, green, black, red in the second row.

In our modified version, we introduced six phases to be comparable with existing datasets of surgical workflow analysis (see Table 1). A phase is a configuration of rings on pegs, and a phase is considered concluded when the final configuration of that phase is reached. The phases are defined as follows:

- *Phase 0*: Random configuration, not corresponding to other phases.
- *Phase 1*: All rings on the green peg.
- *Phase 2*: Two rings on the red peg, and two rings on the blue peg.
- *Phase 3*: All the rings at the center of the board.
- *Phase 4*: One ring in each colored peg.
- *Phase 5*: All rings outside the board.

These configurations create strict sequential dependencies analogous to critical safety steps in surgery (e.g., clipping before cutting), allowing us to benchmark workflow anticipation logic in a deterministic setting.

**Fig. 1:**
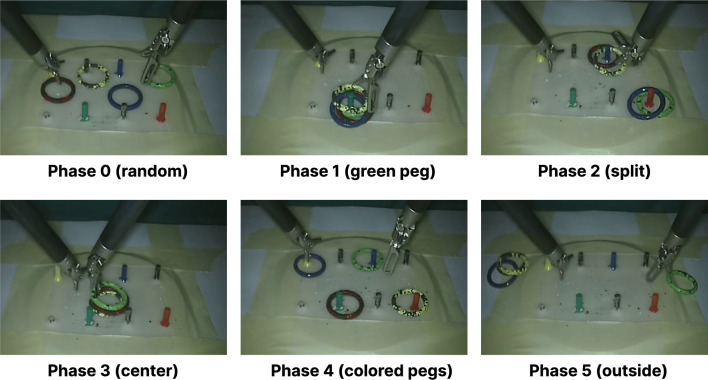
Examples of the phases of the dataset. The first phase is a random configuration that does not match the others

For all phases, the order of movement and placement of colored rings is irrelevant. This choice allows multiple ways to conclude the task, thereby increasing the benchmark’s variability. The dataset comprises 46 videos (720×576 px) at 30 frames per second, with an average duration of 6 min, totalling over 405 k frames. The execution was performed by 15 people without previous experience with the dVRK. In Fig. 1, examples of the phases of the dataset are shown.

### Annotations

For each video, we provide the current phase and the action triplet the robot is executing at a 1-s sampling rate. The phase annotation is the phase index, as indicated in the previous section, yielding more than 11 k labels. Regarding the action triplet, we have 2 instruments, 3 verbs (reach, grasp, release), and 13 targets (each colored ring, each colored peg, the black peg, outside, inside, left arm, and right arm). In total, we have 58 unique triplets and more than 14 k across the dataset (more than phase ones due to bimanual operations like the peg transfer). Each triplet indicates an action lasting between 2 and 6 s.

It’s worth mentioning that CholecT50 [9] includes 100 triplet annotations due to the presence of multiple instruments during the surgery, resulting in a higher number of verbs (10), and therefore more combinations. Nonetheless, the target number is comparable (13 in SPARTAN vs 15 in CholecT50). Data is available here.^1^

### Tasks and metrics

On the SPARTAN benchmark, we propose three tasks: phase recognition, phase anticipation, and action triplet detection, evaluated as follows:

*Phase recognition:* Frame-wise accuracy [23].

*Anticipation:* we report multiple error metrics following [24]: mean absolute error for in-horizon predictions ($\tau ^{\text {gt}} < H$) (*inMAE*), mean absolute error for out-of-horizon predictions ($\tau ^{\text {gt}} = H$) (*oMAE*), weighted average, *wMAE*, defined as $(\text {inMAE} + \text {oMAE})/2$.

*Triplet recognition:* following Nwoye et al. [9], we compute Average Precision (mAP) for verbs and targets (denoted with $mAP_v$ and $mAP_t$), and overall AP as the arithmetic mean of verb and target mAPs ($mAP_{vt}$).

## Method

We address the challenge of multi-task surgical workflow analysis in endoscopic procedures, specifically targeting three complementary objectives: (i) phase recognition, (ii) phase-transition anticipation, and (iii) fine-grained action triplet recognition. Given a temporal sequence of video frames $\mathcal {X} = \{x_t\}_{t=1}^T$, where $x_t \in \mathbb {R}^{H \times W \times 3}$, our model produces a phase classification: $p_t \in \{1, \ldots , C\}$ where C=6 surgical phases. A time-to-next-phase anticipation: $\tau _t \in [0, H]$ where *H* is in minutes and an action triplets: $\langle v_{\text {left}}, t_{\text {left}} \rangle$ and $\langle v_{\text {right}}, t_{\text {right}} \rangle$ for left and right instrument actions, where $v \in \mathcal {V}$ (verbs) and $t \in \mathcal {T}$ (targets).

**Fig. 2:**
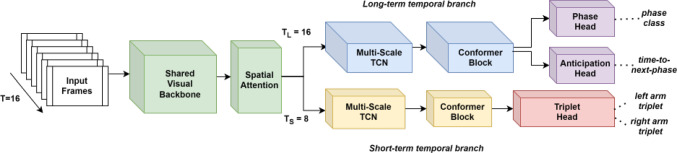
Model architecture of our multi-task approach. Our multi-task architecture employs a shared visual backbone and spatial attention pooling, feeding two distinct temporal paths: a long-term (10–30 s) path for phase/anticipation and a short-term (4–8 s) path for triplet recognition

Due to the temporal scale mismatch between long-horizon phase reasoning and short, fine-grained action triplets, we share a visual backbone across tasks and fork into two temporal pathways: a long-term branch encoding slow changes (which is used for phase and anticipation), and a short-term branch specialized for fast transients (used for triplet/action recognition), using a common feature base [25]. The full architecture diagram is shown in Fig. 2. Let a video clip be denoted by $\textbf{X} \in \mathbb {R}^{B \times T \times 3 \times H \times W}$, with batch size *B*, clip length *T* RGB frames, and spatial resolution H×W. We use a hidden dimensionality *d* for all temporal embeddings. The number of phase classes is *C*, the number of verbs is *V*, and the number of targets is *U*. For temporal scales, we set a long context *T* for phase-related tasks and a short context $T_s$ for triplets. For our experiments, we used d=256, T=16, and $T_s=8$.

Shared visual backbone Each frame is passed through a ResNet backbone (e.g., ResNet-18/50 without the global pooling layer), producing feature maps of size $(B{\cdot }T) \times C_b \times H_b \times W_b$; for ResNet-18, we have $C_b{=}512$. We then apply a lightweight spatial attention pooling (a 1×1 attention mask over the feature map) to obtain one descriptor per frame, resulting in a tensor of shape $(B{\cdot }T) \times C_b$. A frame-level projection head maps these descriptors into the temporal embedding space of dimension *d*, into a sequence $\textbf{F} \in \mathbb {R}^{B \times T \times d}$.

Long-term temporal branch (phase and anticipation) For phase-oriented reasoning, we use all *T* frames available, forming $\mathbf {F_\ell } \in \mathbb {R}^{B \times T \times d}$. The sequence is processed by a multi-scale 1D temporal CNN with exponentially increasing dilations, which captures a hierarchy of temporal dependencies without sacrificing causality. The resulting sequence, still of shape $B \times T \times d$, is then passed through a stack of causal Conformer-style [26] blocks, preserving the temporal dimension and the embedding size *d*. We denote the output by $\textbf{E} \in \mathbb {R}^{B \times T \times d}$. The “current time” representation is the last token $\textbf{e} \in \mathbb {R}^{B \times d}$ (index $T{-}1$). Using that token, we attach two heads: *Phase* and *Anticipation* heads. The first is a final MLP layer that maps $\textbf{e}$ to logits in $\mathbb {R}^{B \times C}$. The latter is a learned query vector attends over the entire $\textbf{E}$ (multi-head attention with a single query per sample), yielding a pooled descriptor in $\mathbb {R}^{B \times d}$. An MLP then produces the regression of time-to-next-phase $\in \mathbb {R}^{B \times C}$ within the horizon interval *H*.

Short-term temporal branch (action triplet) For fine-grained action triplet recognition, we select the most recent $T_s$ embeddings, $\textbf{F}_s \in \mathbb {R}^{B \times T_s \times d}$. The sequence is processed by a shallower multi-scale temporal CNN (again with causal dilations) followed by a Conformer encoder, resulting in $\textbf{E}_s \in \mathbb {R}^{B \times T_s \times d}$. We again take the last token, $\textbf{e}_s \in \mathbb {R}^{B \times d}$, as a causal summary of the short window. From $\textbf{e}_s$ we derive two arm-specific embeddings via independent, deeper MLPs, yielding $\textbf{h}^{L}, \textbf{h}^{R} \in \mathbb {R}^{B \times h_t}$. Each arm branches into two classifiers: verb and target. Consequently, the model emits four logits tensors: left verb (B×V), left target (B×U), right verb (B×V), and right target (B×U).

Train objective The model is optimized in two stages: the first stage focuses solely on phase and anticipation, using cross-entropy for phase recognition and MSE for phase anticipation as $\mathcal {L}_{s1}=\mathcal {L}_{ph}+\mathcal {L}_{ant}$. After training the first stage, we fine-tune the model over the action triplet recognition task using a cross-entropy loss as $\mathcal {L}_{s2}=\mathcal {L}_{s1}+\mathcal {L}_{trip}$.

## Experiments

We conduct comprehensive experiments to evaluate our method on standard benchmarks for surgical workflows. The model is trained in two stages using the AdamW optimizer with a weight decay of $2 \times 10^{-4}$. In the first stage, the network is trained for phase recognition/anticipation over 100 epochs. The second stage fine-tunes the model on the action triplet task for an additional 100 epochs. We implemented the model in PyTorch on a single RTX 5090 (for inference) and an A100 (for training). The learning rate is set to $1 \times 10^{-4}$ for Stage 1 and reduced to $5 \times 10^{-5}$ during fine-tuning. A 5-epoch linear warm-up is applied, followed by cosine annealing for learning rate scheduling.

For experiments on Cholec80, HeiChole, and AutoLaparo, we set T=30 for both phase recognition and anticipation, while for CholecT50, we use $T_s=8$. For SPARTAN, we set T=16 and $T_s=8$. We evaluate the proposed model on Cholec80 [1], HeiChole [21], and AutoLaparo [22] for surgical phase recognition and anticipation, and on CholecT50 [9] for surgical action triplet recognition. Performance is measured using standard metrics (see Section Tasks and metrics). Results w.r.t. literature are reported in Table 2 for phase and anticipation, and Table 3 for action triplet detection.

**Table 2:** Comparison of phase and anticipation results on different literature datasets

Method	Latency	Cholec80	HeiChole	AutoLaparo	oMAE	wMAE	inMAE
SKiT [8]	7.0	**93**.**4**	–	**82**.**9**	–	–	–
MuST [7]	98.8	92.0	**82**.**0**	–	–	–	–
SUPR-GAN [15]	*N/A*	82.3	–	–	–	–	–
SWAG [16]	31.2	88.3	–	73.3	0.34	0.80	1.26
IIA-Net [14]	*N/A*	–	–	–	0.52	0.85	1.17
Ours	15.0	89.8 ± 0.8	80.6 ± 2.1	78.5 ± 1.5	**0**.**31** ± 0.05	**0**.**79** ± 0.06	1.27 ± 0.1

Bold type indicates the best-performing value in each column (i.e., the highest value for ascending metrics and the lowest value for descending metrics)

The column labeled with dataset name refers to the phase recognition accuracy. The anticipation metrics, *oMAE*, *wMAE*, and *inMAE*, are calculated at H=5 min and refer to the Cholec80 dataset. Latency is in milliseconds; values come from papers or implementations, when available, *N/A* otherwise. Errors (±), when available, indicate the standard deviation computed over three independent runs

**Table 3:** Comparison with triplet on CholecT50

Method	Latency	$\text {mAP}_{\text {i}}$	$\text {mAP}_{\text {v}}$	$\text {mAP}_{\text {t}}$	$\text {mAP}_{\text {iv}}$	$\text {mAP}_{\text {it}}$	$\text {mAP}_{\text {ivt}}$
Rendezvous [9]	52.36	89.4 ± 2.0	60.4 ± 2.8	40.3 ± 2.2	34.5 ± 2.8	31.8 ± 1.0	29.4 ± 2.5
RiT [10]	25.7	88.6 ± 2.6	64.0 ± 2.5	43.4 ± 1.4	38.3 ± 3.5	36.9 ± 1.0	29.7 ± 2.6
TDN [11]	55.1	91.2 ± 1.9	65.3 ± 2.8	43.7 ± 1.6	-	-	33.8 ± 2.5
MT4MTL-KD [13]	*N/A*	93.1 ± 2.1	71.8 ± 3.4	48.8 ± 3.8	44.9 ± 2.4	43.1 ± 2.0	37.1 ± 0.5
DiffTriplet [12]	59.17	95.3 ± 1.4	70.9 ± 0.8	53.1 ± 2.6	46.4 ± 4.1	48.2 ± 1.8	40.3 ± 2.5
Ours	15.0	85.9 ± 1.8	62.3 ± 2.1	42.3 ± 2.8	41.4 ± 1.4	35.8 ± 2.1	34.3 ± 1.8

Latency is in milliseconds; values come from papers or implementations, when available, *N/A* otherwise

**Table 4:** Results on SPARTAN of our model, comparing phase recognition, anticipation at H=2 min, and action triplet results

Method	Acc.	MAE	inMAE	oMAE	wMAE	$\text {AP}_{v}$	$\text {AP}_{t}$	$\text {AP}_{vt}$
SKiT* [8]	**0**.**66** ± 1.2	–	–	–	–	–	–	–
MuST [7]	0.64 ± 0.9	0.46 ± 0.8	0.52 ± 0.7	0.41 ± 0.7	0.46 ± 0.7	–	–	–
SWAG* [16]	0.49 ± 1.0	0.45 ± 1.1	0.49 ± 1.0	0.44 ± 1.2	0.47 ± 1.0	–	–	–
TDN* [11]	–	–	–	–	–	0.45 ± 0.2	0.38 ± 1.0	0.42 ± 0.6
RiT [10]	–	–	–	–	–	**0**.**44** ± 0.2	0.37 ± 0.8	0.41 ± 0.5
Ours	0.63 ± 0.8	**0**.**35** ± 1.0	**0**.**42** ± 0.8	**0**.**27** ± 0.3	**0**.**34** ± 0.5	**0**.**44** ± 0.2	**0**.**36** ± 1.4	**0**.**40** ± 0.9

Bold type indicates the best-performing value in each column (i.e., the highest value for ascending metrics and the lowest value for descending metrics)

Given the presence of only two instruments, we are reporting only $\text {AP}_{v}$, $\text {AP}_{t}$, and $\text {AP}_{vt}$. “Acc.” is phase accuracy. Some methods were omitted due to code unavailability. *Methods re-implemented following papers, and adapted for this benchmark. Errors (±) indicate the standard deviation computed over three independent runs

Regarding phase recognition, we show in Table 2 that we achieve results comparable to the state of the art, with real-time latency (second lowest) and a throughput of up to 60 FPS. In the phase anticipation benchmarks, we achieve better results than current alternative approaches. The results at H=2 and H=3 min were similar in terms of values but were omitted due to space constraints. We believe the H=5 min are more significant when we consider long videos like those of cholecystectomy, whereas with shorter time horizons, errors are usually smaller overall. While SOTA models achieve marginally higher accuracy in long-horizon anticipation, our method offers a significant speedup, which is critical for the target application of real-time robotic control. When considering action triplets, as shown in Table 3, we can see that our model is extremely faster (∼60 FPS) with respect to other literature approaches (∼20 FPS), making it viable for online robotic control, while being comparable to state-of-the-art in terms of average precision. For instance, recent methods like DiffTriplet [12] outperform previous methods, but at the cost of a higher computational burden that requires powerful hardware. The CholecT50 performance gap reflects a trade-off between speed and accuracy. We present a real-time baseline for joint phase, anticipation, and triplet prediction, acknowledging that larger models achieve higher accuracy only by sacrificing latency.

While specialized offline architectures may achieve higher accuracy on individual tasks (e.g., pure anticipation), they often sacrifice real-time capability. Our unified framework prioritizes inference speed to meet the dVRK’s 30Hz control loop requirement. The lower scores on Cholec80 compared to offline SOTA reflect the latency-accuracy trade-off required for transitioning from video analysis to robotic control.

Finally, we show the results of our approach on the SPARTAN dataset. We split the 46 videos into 41 for training and 5 for testing set. As shown in Table 4, the results are encouraging; however, the task is far from solved. The complexity introduced by the dataset, with multiple users utilizing the robotic platform, bimanual operations, and varying movement dynamics, results in high variability in the task.

We intentionally exclude instrument detection to ensure our model remains generalizable and independent of external annotations. While this choice makes the task more challenging and impacts current performance, it highlights the need for robust reasoning, a gap our benchmark aims to fill. The lower scores on the phase and anticipation tasks stem from the deliberate lack of unique visual cues and the high variability of valid phase configurations, which ensure the problem remains non-trivial. At the same time, the limited number of instruments makes it easier to find the correct action triplet, but the higher manipulations (introduced in the dataset to increase variability) make the task far from trivial, as evidenced by the maximum AP obtained on the benchmark by several methods. In fact, $AP_i$ for Table 3 is the only one particularly high (over 85%), meaning the task of identifying the correct instrument is already a relatively easy task to address, thanks to visual clues of the instruments themselves, while other tasks, verb and targets, remain critical and require advanced reasoning. Lastly, the observed variance in anticipation metrics correlates with inter-subject variability in task execution speed. Future iterations could mitigate this by incorporating user-specific velocity tokens or intent embeddings.

### Action-level complexity analysis

To address potential concerns that SPARTAN’s abstracted visual domain might yield a trivial or deterministic reasoning task, we conducted a quantitative analysis of action-level complexity, comparing SPARTAN to the widely used CholecT50 dataset. Using standard information-theoretic and distributional metrics computed directly from ground-truth annotations, we assessed the underlying reasoning difficulty of both benchmarks. Our analysis reveals that SPARTAN exhibits substantially higher action-level uncertainty. The conditional action entropy [27], which measures the residual uncertainty of an action given the current workflow phase, is 4.67 bits for SPARTAN (single-arm) compared to 2.85 bits for CholecT50. This indicates that SPARTAN actions remain far less predictable even when the phase is known. Consistently, Top-1 coverage [27] shows that trivially predicting the most frequent action per phase yields 49.5% success in CholecT50 but only 12.0% in SPARTAN, confirming a much flatter and more diverse per-phase action distribution.

Importantly, SPARTAN also exhibits higher inter-phase action overlap: the average Jaccard similarity [27] between phase-conditioned action vocabularies is 0.575 in SPARTAN versus 0.256 in CholecT50. This means many of the same actions appear across multiple phases, so phase context alone is insufficient to resolve the correct action. Consequently, models must rely more on fine-grained visual-state reasoning rather than on phase priors.

Finally, SPARTAN introduces simultaneous bimanual coordination, encompassing 235 distinct dual-arm joint states, with both arms active 9.1% of the time. This multi-agent coordination requirement is absent from single-instrument surgical benchmarks, further increasing the complexity of reasoning. Overall, despite simplified visual appearance, SPARTAN provides a high-entropy, multi-agent action space that constitutes a challenging benchmark for surgical workflow reasoning. Full metric definitions and detailed tables are provided in the supplementary material.

## Conclusions

In this work, we presented SPARTAN, a novel benchmark and unified framework for real-time surgical workflow analysis that jointly performs *surgical phase recognition*, *surgical phase anticipation*, and *surgical action triplet recognition*. Our approach bridges the gap between high-level procedural understanding and low-level, actionable perception: an essential step toward autonomous and context-aware surgical robotics. The SPARTAN benchmark, based on a reproducible phase-based Peg-and-Ring setup, provides dense temporal annotations of both workflow phases and dual-arm action triplets, thereby enabling systematic research on closed-loop perception under realistic robotic constraints. Through extensive experiments, we demonstrated that the proposed architecture achieves comparable state-of-the-art performance, while showing the challenges yet to be solved in combining phase/anticipation with action triplet recognition, motivating our work.

While SPARTAN lacks the visual complexity of biological tissue, its controlled environment serves as a ‘sandbox’ to isolate and evaluate algorithmic capabilities (specifically long-horizon reasoning and fine-grained triplet recognition) without the confounding noise inherent in clinical data. We believe SPARTAN provides a foundation for the next generation of intelligent surgical systems capable of understanding, anticipating, and responding to dynamic intraoperative contexts in real time. The performance gap between SPARTAN and clinical datasets highlights the challenge of visual domain generalization, while establishing SPARTAN as a necessary testbed for validating logical workflows. Future work will explore extending the benchmark to more complex surgical scenarios, integrating kinematic data, and coupling perception with autonomous control policies. Ultimately, we envision SPARTAN as a catalyst for safe, transparent, and fully autonomous robotic surgery.

## Supplementary Information

Below is the link to the electronic supplementary material.

Supplementary file 1 (pdf 659 KB)

## Footnotes

1 https://gitlab.com/altairLab/spartan.
