# Supplementary material for: Spartan: surgical peg-and-ring triplet and workflow anticipation benchmark
Source: Int J Comput Assist Radiol Surg. 2026 May 6;21(6):1187–95. doi: 10.1007/s11548-026-03688-y (PMC13451315; doi:10.1007/s11548-026-03688-y)
Supplement: Supplementary file 1 [file 11548_2026_3688_MOESM1_ESM.pdf]

SPARTAN: Surgical Peg-And-Ring Triplet and Workflow Anticipation Benchmark

Supplementary Material

Abstract

This additional material provides comprehensive documentation of the SPARTAN dataset, addressing key aspects of the benchmark that complement the main paper. We present: (1) detailed dataset statistics and temporal characteristics, (2) the complete annotation protocol for surgical phases and action triplets, (3) bimanual coordination patterns unique to our dataset, (4) anticipation ground truth computation methodology, and (5) extensive comparisons with related benchmarks. These materials demonstrate the dataset's value for advancing research on anticipating surgical workflows and recognising action triplets.

Contents

1 Dataset Overview
- 1.1 Recording Setup
- 1.2 Dataset Statistics

2 Annotation Protocol
- 2.1 Surgical Phase Definitions
- 2.2 Action Triplet Vocabulary
- 2.3 Annotation Density

3 Temporal Characteristics
- 3.1 Phase Duration Analysis
- 3.2 Key Temporal Observations

4 Bimanual Action Coordination
- 4.1 Coordination Patterns
- 4.2 Coordination Statistics

5 Anticipation Ground Truth
- 5.1 Time-to-Phase Computation
- 5.2 Anticipation Visualization
- 5.3 Anticipation Units

6 Sample Frames

7 Benchmark Comparison
- 7.1 Comparison with Related Datasets
- 7.2 Detailed Comparison
- 7.3 Action-Level Complexity Analysis
  - 7.3.1 Triplet Ontology
  - 7.3.2 Metric Definitions
  - 7.3.3 Discussion

1 Dataset Overview

The SPARTAN dataset consists of **46 videos** capturing bimanual peg-and-ring transfer tasks performed by multiple participants with varying skill levels. This section provides a comprehensive overview of the dataset structure and statistics.

1.1 Recording Setup

All videos were recorded using the dVRK Classic endoscopic camera, capturing the complete workspace at 30 frames per second (fps) with a resolution of 720×576 pixels. The recording environment includes:

- A standardised pegboard with 4 colored pegs (yellow, green, red, blue) plus 1 grey anchor peg
- 4 colored rings (yellow, green, red, blue) to be manipulated
- Consistent lighting conditions across all recordings
- Clear visibility of both the participant's hands and all workspace elements

1.2 Dataset Statistics

Table 1 summarises the key statistics of the SPARTAN dataset.

Table 1: SPARTAN Dataset Statistics Summary

**Property**	**Value**
Total Videos	46
Total Duration	$\sim$3.6 hours
Mean Video Duration	4.7 min ($\pm$1.4 min)
Frames per Video (original)	$\sim$7,000
Frames per Video (1 fps)	$\sim$280
Number of Phases	6
Number of Triplet Verbs	3 (+null)
Number of Triplet Subjects	2 (+null)
Number of Triplet Targets	13 (+null)
Train/Validation Split	41/5 videos
Validation Videos	v01, v13, v15, v22, v27

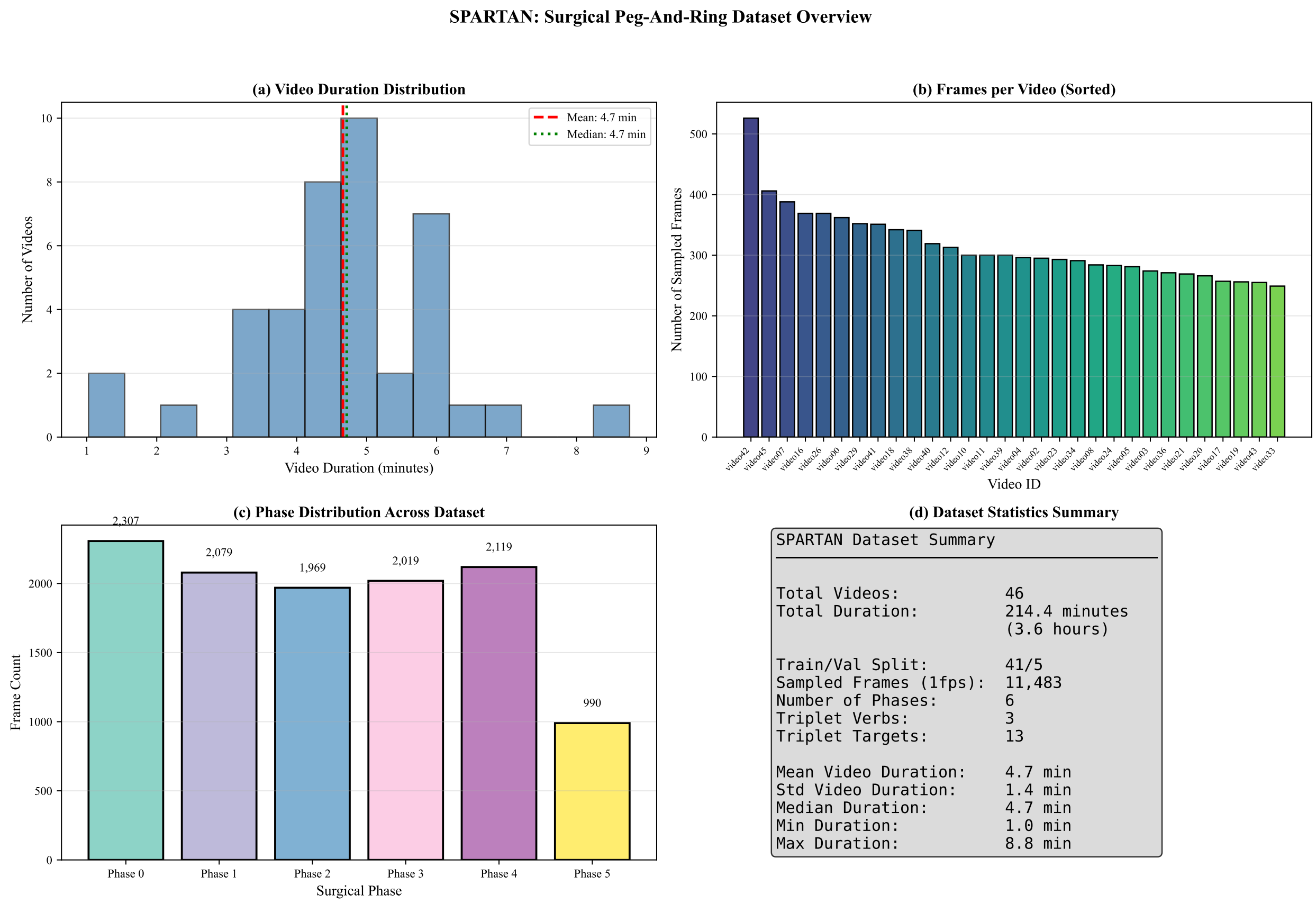

Figure 1: **Dataset Overview.** (a) Video duration distribution across the dataset, showing mean and median values. (b) Number of sampled frames per video, sorted by count. (c) Distribution of surgical phases across all annotated frames. (d) Summary of key dataset statistics.

2 Annotation Protocol

This section details the multi-level annotation protocol employed in SPARTAN, including surgical-phase labels and action-triplet annotations for both arms.

2.1 Surgical Phase Definitions

The peg-and-ring task is structured into **6 sequential surgical phases** (P0-P5), each representing a distinct goal state of the ring configuration:

P0: Initial Configuration - Random non-final arrangement of rings on the pegboard
P1: All on Green - All four rings placed on the green peg
P2: Half Red/Half Blue - Two rings on red peg, two on blue peg
P3: All in Center - All rings in center area without pegs
P4: One per Peg - One ring on each of the four colored pegs (Y/G/R/B)
P5: All Outside - All rings placed outside the pegboard circle

Each video transitions through all phases in sequence, with varying durations depending on participant skill and strategy.

2.2 Action Triplet Vocabulary

Following the triplet representation introduced by CholecT50 [1], we define action triplets as $\langle \text{verb}, \text{subject}, \text{target} \rangle$ tuples. Our vocabulary is specifically designed for bimanual manipulation; Table 2 details the components of our action triplet vocabulary.

Table 2: Action Triplet Vocabulary Components

**Verbs (3)**	**Subjects (2)**	**Targets (13)**
reach	left_arm	*Pegs:* red, green, blue, yellow, grey
grasp	right_arm	*Rings:* red, green, blue, yellow
release		*Locations:* center, outside, left_arm, right_arm

This yields a theoretical vocabulary of $3 \times 2 \times 13 = 78$ unique triplets; however, in practice, approximately 60 triplets are observed due to task constraints.

2.3 Annotation Density

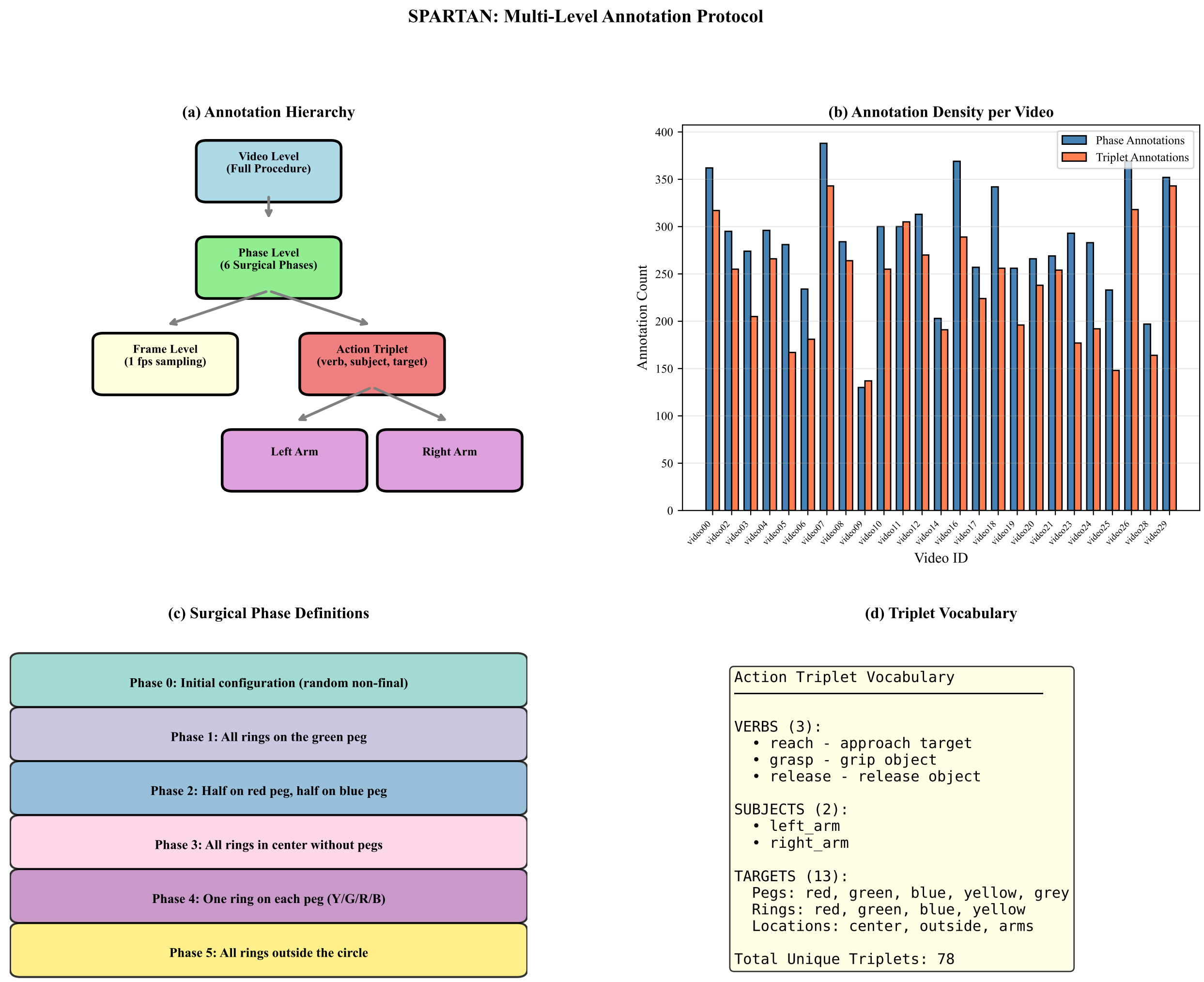

Figure 2: **Annotation Protocol.** (a) Hierarchical structure of multi-level annotations from video-level to frame-level to action triplets. (b) Annotation density comparison showing phase and triplet annotation counts per video. (c) Surgical phase definitions with colour coding. (d) Complete triplet vocabulary breakdown.

Annotations are provided at **1-second intervals** (1 fps sampling), resulting in:

- **Phase annotations:** Every sampled frame has a phase label
- **Triplet annotations:** Dual-arm annotations at each second, capturing simultaneous bimanual actions
- **Anticipation labels:** Time-to-next-phase computed for all 6 phases at each frame

3 Temporal Characteristics

Understanding the temporal dynamics of surgical procedures is crucial for anticipation tasks. This section analyses the temporal properties of the SPARTAN dataset.

3.1 Phase Duration Analysis

Figure 3 presents a comprehensive analysis of phase durations and transition patterns.

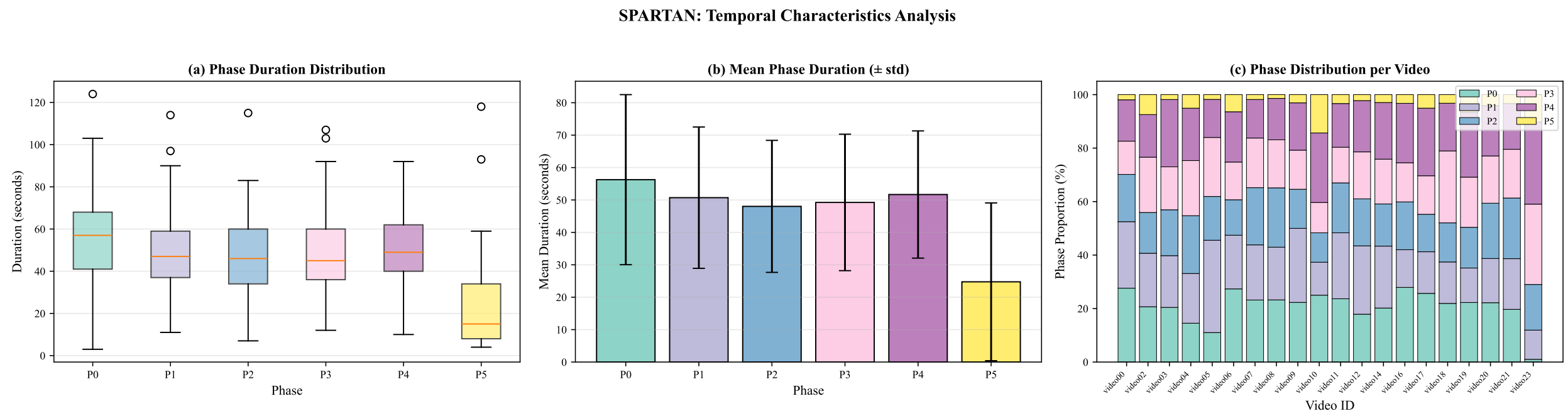

Figure 3: **Temporal Characteristics Analysis.** (a) Phase duration distributions shown as box plots. (b) Mean phase duration with standard deviation error bars. (c) Stacked bar chart showing phase proportion distribution per video.

3.2 Key Temporal Observations

- **Phase Duration Variability:** Phase durations vary significantly across videos, reflecting different participant strategies and skill levels. The initial configuration phase (P0) typically has the shortest duration, while manipulation phases (P1-P4) show greater variability.
- **Sequential Transitions:** The transition probability matrix confirms the expected sequential workflow, with high probabilities along the diagonal offset. Occasional non-sequential transitions occur when participants correct errors.
- **Workflow Complexity:** Videos contain 6-12 distinct phase segments on average, indicating that participants may revisit certain configurations during task execution.

4 Bimanual Action Coordination

A unique aspect of the SPARTAN dataset is its focus on **bimanual coordination**, capturing simultaneous actions of both left and right arms.

4.1 Coordination Patterns

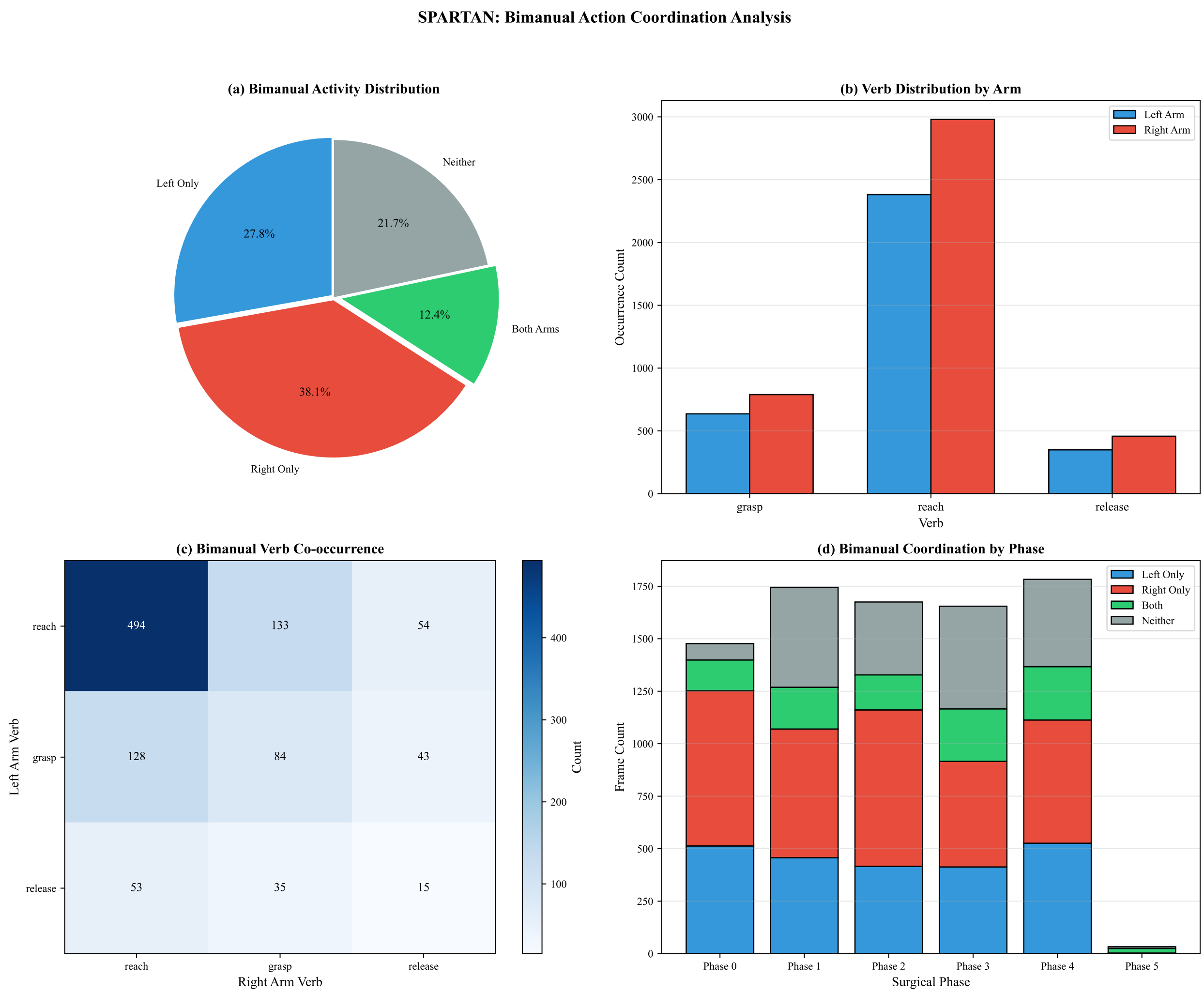

Figure 4: **Bimanual Coordination Analysis.** (a) Distribution of activity states showing when one arm, both arms, or neither is active. (b) Verb distribution comparison between left and right arms. (c) Bimanual verb co-occurrence heatmap showing which verbs are performed simultaneously. (d) Coordination patterns across different surgical phases.

4.2 Coordination Statistics

Table 3 summarises the bimanual coordination patterns observed in the dataset.

Table 3: Bimanual Coordination Statistics

Activity State	Percentage	Description
Both arms active	12%	Simultaneous bimanual manipulation
Left arm only	28%	Unimanual left arm action
Right arm only	38%	Unimanual right arm action
Neither active	22%	Transition/idle states

These statistics demonstrate that bimanual actions constitute a significant portion of the task, justifying the need for

dual-arm action recognition approaches.

5 Anticipation Ground Truth

This section describes the methodology for computing anticipation ground truth labels, which are essential for training and evaluating surgical workflow anticipation models.

5.1 Time-to-Phase Computation

For each frame t in video v, we compute the anticipation values as a 6-dimensional vector $\mathbf{a}_t \in \mathbb{R}^6$, where each component represents the time until the corresponding phase begins (or 0 if that phase has already started):

$$a_t^{(p)} = \begin{cases} \tau_p - t & \text{if phase } p \text{ has not yet started at frame } t \\ 0 & \text{if phase } p \text{ is current or past} \end{cases} \tag{1}$$

where τ_p is the frame index at which phase p begins.

5.2 Anticipation Visualization

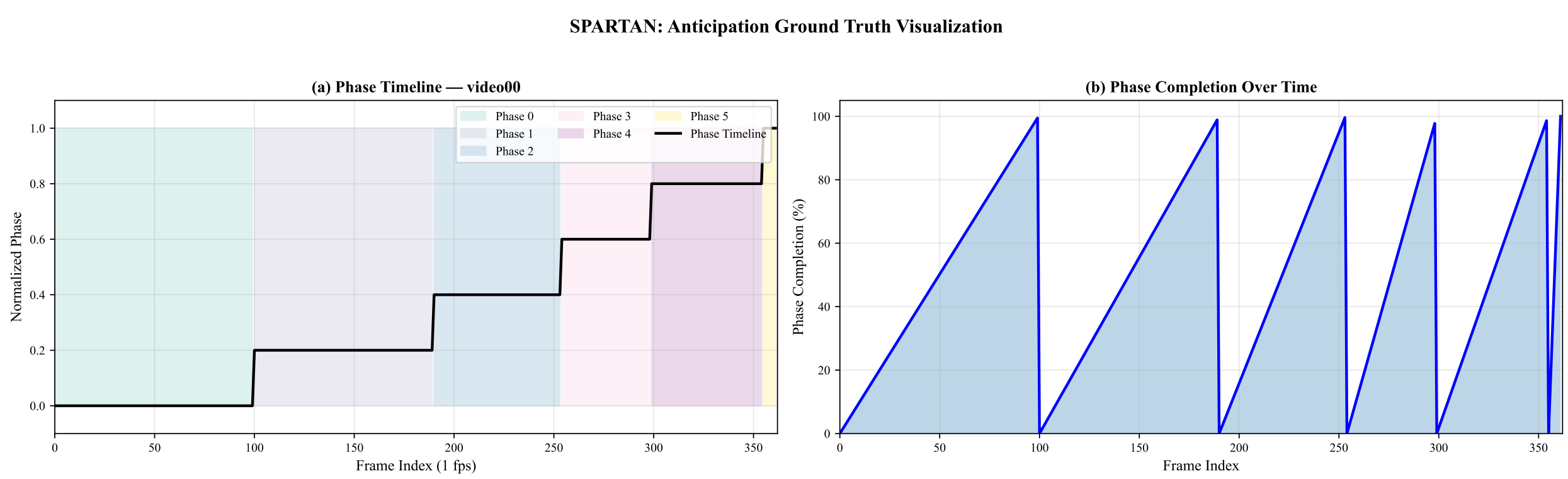

Figure 5: **Anticipation Ground Truth Visualisation.** (a) Phase timeline for a sample video showing phase boundaries and temporal progression. (b) Phase completion percentage over time showing workflow progress.

5.3 Anticipation Units

Anticipation values can be expressed in multiple units depending on the application:

- **Frames:** Raw frame counts (1 fps sampling)
- **Seconds:** Time in seconds (equivalent to frames at 1 fps)
- **Minutes:** Time in minutes for longer-horizon anticipation

Our PyTorch dataloader supports configurable time units through the `time_unit` parameter.

6 Sample Frames

Figure 6 shows representative frames from each surgical phase, illustrating the visual characteristics and ring configurations.

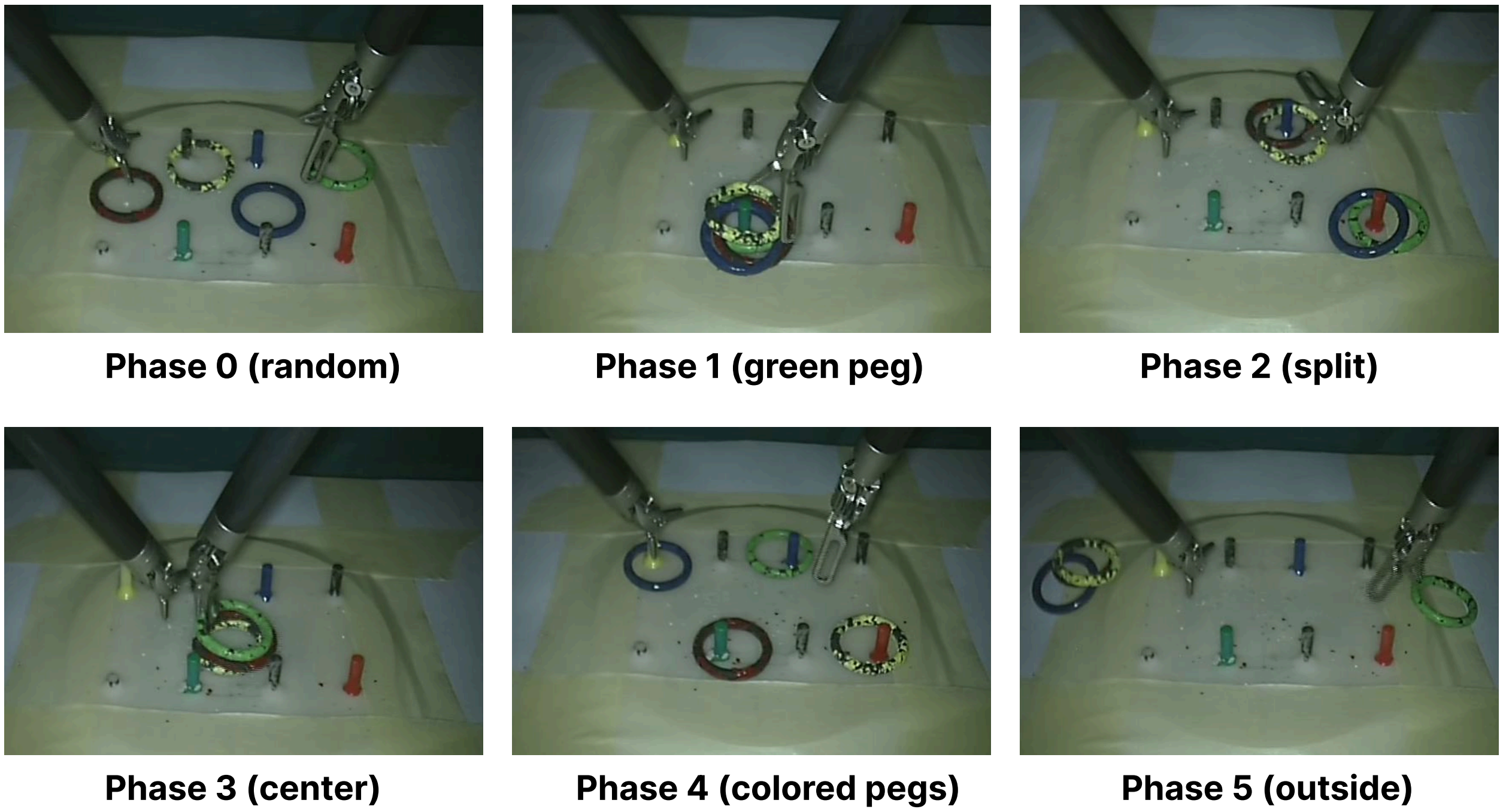

Figure 6: **Sample Frames.** Representative frames from each of the 6 surgical phases, showing the characteristic ring configurations. Each phase has distinct visual features that models must learn to recognise and anticipate.

7 Benchmark Comparison

We compare SPARTAN with existing surgical workflow datasets to highlight its unique contributions.

7.1 Comparison with Related Datasets

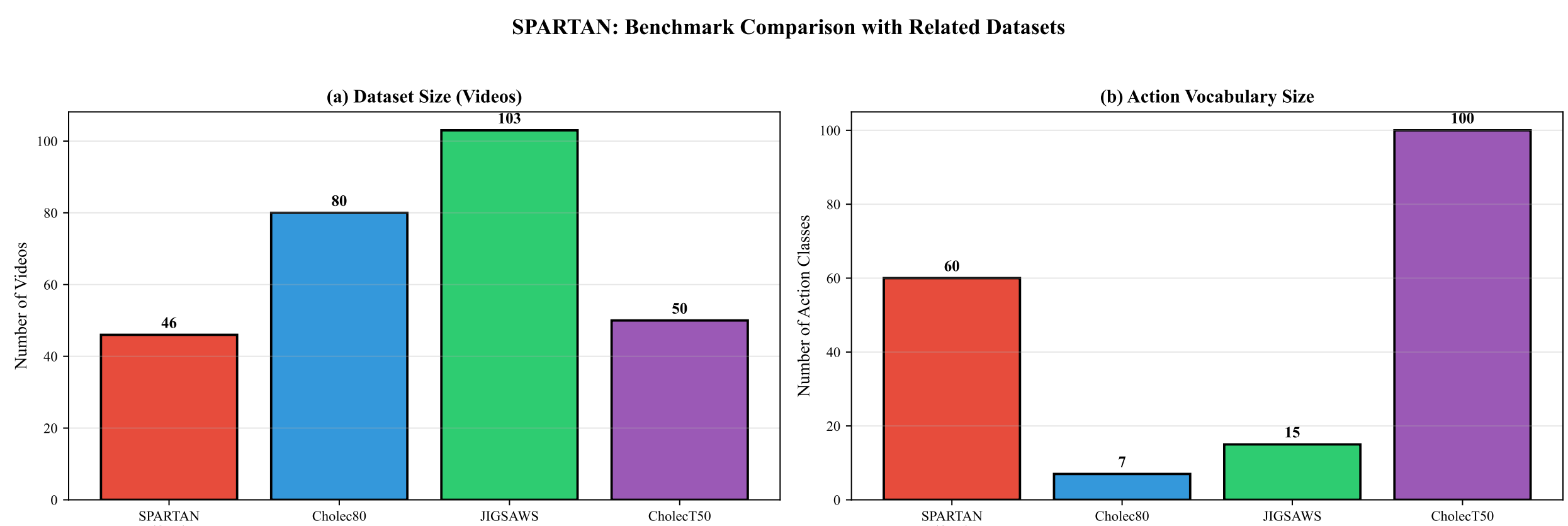

Figure 7: **Benchmark Comparison.** (a) Number of videos in each dataset. (b) Action vocabulary size comparison showing SPARTAN's comprehensive action space.

7.2 Detailed Comparison

Table 4 provides a comprehensive quantitative comparison between SPARTAN and existing surgical workflow datasets.

Table 4: Comparison with Related Surgical Workflow Datasets

Dataset	Videos	Hours	Phases	Actions	Triplets	Anticipation	Bimanual
Cholec80	80	50	7	-	×	×	×
JIGSAWS	103	3.3	-	15	×	×	×
CholecT50	50	40	7	-	✓	×	×
Heichole	33	30	7	-	×	×	×
**SPARTAN (Ours)**	**46**	**7.5**	**6**	**60+**	✓	✓	✓

7.3 Action-Level Complexity Analysis

A common concern with standardised robotic tasks is that they may be *deterministic*: given a workflow phase, the action could be trivially predictable. We investigate this quantitatively by comparing the action-level complexity of SPARTAN with CholecT50 [1], the most widely used surgical action-triplet benchmark. All metrics below are computed directly from the annotation data of both datasets; no simulation or synthetic data is involved.

7.3.1 Triplet Ontology

CholecT50. Each frame is annotated with one or more *Instrument-Verb-Target* (IVT) triplets drawn from a closed vocabulary of $|\mathcal{I}| = 6$ instruments, $|\mathcal{V}| = 10$ verbs, and $|\mathcal{T}| = 15$ targets, yielding 100 defined triplet classes (94 non-null, of which 94 are observed in the data). Frames are assigned to one of 7 surgical phases. Across 50 videos and $100,863$ frames, 47.4% of frames carry more than one simultaneous IVT triplet (mean 1.39 per frame), reflecting multi-instrument use.

SPARTAN. SPARTAN annotates a bimanual peg-and-ring task with *Verb-Subject-Target* triplets where each arm acts independently: $|\mathcal{V}| = 3$ observed verbs (`reach`, `grasp`, `release`), $|\mathcal{S}| = 2$ subjects (`left_arm`, `right_arm`), and $|\mathcal{T}| = 13$ observed targets (5 pegs, 4 rings, and spatial/arm references). The two arms act simultaneously, producing a *dual-arm joint state* at every time step. Across 33 annotated videos and $5,123$ annotated seconds ($5,869$ single-arm annotation instances), we observe 58 distinct single-arm triplets and 235 distinct dual-arm joint states. Both arms are simultaneously active in 9.1% of annotated time. The task spans 6 workflow phases.

7.3.2 Metric Definitions

We employ the following information-theoretic and distributional metrics [2]:

1. Conditional Action Entropy measures the residual uncertainty in the action *after* the phase is known:

$$H(\mathcal{A} \mid \mathcal{P}) \;=\; \sum_{p \in \mathcal{P}} P(p)\; H(\mathcal{A} \mid \mathcal{P}{=}p), \quad H(\mathcal{A} \mid \mathcal{P}{=}p) = -\sum_{a} P(a \mid p)\, \log_2 P(a \mid p). \tag{2}$$

Interpretation: $H = 0$ means the phase fully determines the action (deterministic/scripted); higher H means a model must inspect the visual input to resolve the action, even when the phase is known.

2. Normalised Conditional Entropy removes the bias towards larger vocabularies:

$$\hat{H} \;=\; \frac{H(\mathcal{A} \mid \mathcal{P})}{\log_2 |\mathcal{A}_{\text{obs}}|}, \tag{3}$$

where $|\mathcal{A}_{\text{obs}}|$ is the number of *observed* action types. $\hat{H} \in [0, 1]$: values near 1 indicate that, per phase, actions are spread almost uniformly over the entire observed vocabulary.

3. Gini Coefficient of the per-phase action distribution:

$$G_p = \frac{2\sum_{i=1}^{n} i \cdot x_{(i)}}{n\sum_{i=1}^{n} x_{(i)}} - \frac{n+1}{n}, \tag{4}$$

where $x_{(1)} \leq \ldots \leq x_{(n)}$ are the sorted action counts in phase p. *Interpretation:* $G = 0$ means all actions are equally frequent (maximum diversity); $G \to 1$ means a single action dominates (minimum diversity). We report the mean across phases: lower $\bar{G}$ implies a harder prediction task.

4. Top-k Coverage is the fraction of instances captured by the k most frequent actions within a phase. *Interpretation:* High top-1 coverage means a naïve "always predict the most common action" baseline already performs well; low coverage indicates an informative long tail of less-frequent actions.

5. Effective Branching Factor (BF) counts actions whose per-phase relative frequency exceeds 1%. *Interpretation:* Larger BF means more actions are viable in a given phase, increasing prediction difficulty.

6. Inter-Phase Jaccard Index measures the overlap between the action vocabularies of two phases:

$$J(p_i, p_j) = \frac{|\mathcal{A}_{p_i} \cap \mathcal{A}_{p_j}|}{|\mathcal{A}_{p_i} \cup \mathcal{A}_{p_j}|}. \tag{5}$$

We report the average over all off-diagonal phase pairs. *Interpretation:* High Jaccard means many actions are shared across phases—the phase label alone is insufficient to discriminate actions. Low Jaccard means actions are phase-specific.

7. Transition Entropy captures temporal action dynamics:

$$\bar{H}_{\text{trans}} = \frac{1}{|C|}\sum_{c \in C} H(\mathcal{A}_{t+1} \mid \mathcal{A}_t = c), \tag{6}$$

where C is the set of observed current-action contexts. *Interpretation:* $\bar{H}_{\text{trans}} = 0$ means the current action fully determines the next one (rigid linear workflow); higher values indicate genuine branching in action sequences.

Additionally, Table 6 summarises the per-video action diversity across both datasets.

A detailed breakdown of per-phase triplet statistics is provided in Table 7 for CholecT50 and Table 8 for SPARTAN.

7.3.3 Discussion

Table 5 yields the following key observations:

1. **SPARTAN exhibits higher conditional entropy than CholecT50.** At the single-arm level, $H(\mathcal{A} \mid \mathcal{P})_{\text{SPARTAN}} = 4.67$ bits versus 2.85 bits for CholecT50, meaning that for each phase there is *more* residual uncertainty in the action. The dual-arm joint entropy rises further to 5.11 bits. This conclusively refutes the hypothesis that SPARTAN is deterministic.

2. **Normalised entropy confirms the gap.** Accounting for vocabulary size, $\hat{H}^{\text{1-arm}}_{\text{SPARTAN}} = 0.796$ and $\hat{H}_{\text{CholecT50}} = 0.435$. SPARTAN thus uses a *larger fraction* of its observed action space per phase, making it strictly harder in normalised terms.

3. **Target entropy is the main driver in SPARTAN.** $H(\text{Target} \mid \mathcal{P})_{\text{SPARTAN}} = 2.73$ bits vs. 1.88 bits in CholecT50. The multiplicity of visually similar ring and peg targets generates discrimination difficulty not present in surgical scenes where a single anatomical structure dominates each phase.

4. **Lower concentration in SPARTAN.** The average Top-1 coverage in SPARTAN is 0.120, meaning the most frequent action covers only 12.0% of each phase's instances compared with 49.5% in CholecT50. The lower Gini coefficient ($\bar{G}_{\text{SPARTAN}} = 0.486$ vs. $\bar{G}_{\text{CholecT50}} = 0.822$) confirms that actions are more uniformly distributed.

Table 5: Objective comparison of action-level complexity. All values are computed from real annotations. *1-arm* refers to single-arm SPARTAN triplets; *2-arm* to the joint bimanual state. Arrows indicate the direction that signals higher complexity.

**Metric**	**CholecT50**	**SPARTAN (1-arm)**	**SPARTAN (2-arm)**
Ontology			
Workflow phases	7	6	6
Videos	50	33	33
Annotated time-steps	100,863	5,123	5,123
Observed triplet types $\lvert\mathcal{A}_{\text{obs}}\rvert$	94	58	235
Conditional Entropy (↑ = harder)			
$H(\mathcal{A} \mid \mathcal{P})$ [bits]	2.85	4.67	5.11
$\hat{H}$ (normalised)	0.435	0.796	0.649
$H(\text{Verb} \mid \mathcal{P})$ [bits]	1.81	1.19	-
$H(\text{Target} \mid \mathcal{P})$ [bits]	1.88	2.73	-
Concentration & Uniformity			
Avg. Gini coefficient $\bar{G}\downarrow$	0.822	0.486	0.592
Avg. Top-1 coverage ↓	0.495	0.120	0.112
Avg. Top-3 coverage ↓	0.768	0.304	-
Avg. branching factor (BF) ↑	8.6	25.2	24.0
Cross-Phase & Temporal			
Avg. inter-phase Jaccard	0.256	0.575	0.236
$\bar{H}_{\text{trans}}$ [bits] ↑	1.412	2.624	0.981
Multi-Agent			
Simultaneous agents	≤ 3 inst.	2 arms (always)	
Dual-arm coordinated [%]	-	9.1%	

Table 6: Per-video action diversity (number of unique triplet types observed per video).

	**CholecT50**	**SPARTAN (1-arm)**	**SPARTAN (2-arm)**
Mean ± Std	26.5 ± 5.5	32.4 ± 2.9	38.8 ± 8.7
Min-Max	17-41	27-37	27-61

5. **Higher inter-phase action overlap in SPARTAN** ($J = 0.575$ vs. 0.256) means that many of the same triplets (e.g., `reach` toward different targets) appear across multiple phases. Consequently, the phase label alone is *insufficient* to predict the action; a model must reason about the visual state to resolve the correct target.

6. **Bimanual coordination introduces a qualitatively new dimension.** With 235 observed joint states and 9.1% of time with both arms active, SPARTAN uniquely requires predicting *simultaneous, coordinated* multi-agent actions, a capability not tested by any single-instrument surgical benchmark.

Table 7: CholecT50 per-phase triplet statistics (non-null triplets only).

ID	Phase	Uniq	H [bit]	G	BF	Top triplet (%)
0	Preparation	17	2.26	0.77	7	`grasper,retract,gallbladder` (57%)
1	Calot Triangle Dissection	68	3.23	0.88	8	`grasper,retract,gallbladder` (38%)
2	Clipping and Cutting	33	2.85	0.82	10	`grasper,retract,gallbladder` (45%)
3	Gallbladder Dissection	47	2.40	0.90	7	`hook,dissect,gallbladder` (46%)
4	Gallbladder Packaging	16	2.07	0.80	7	`grasper,grasp,specimen_bag` (55%)
5	Cleaning and Coagulation	36	3.09	0.79	13	`grasper,retract,liver` (41%)
6	Gallbladder Retraction	16	1.93	0.80	8	`grasper,grasp,specimen_bag` (64%)

Table 8: SPARTAN per-phase action statistics from real annotations. *1-arm* = single manipulator triplets; *2-arm* = joint bimanual state.

ID	Phase	1-arm		2-arm		G	BF
		Uniq	H	Uniq	H		
0	Idle / Preparation	40	4.57	64	4.81	0.54	27
1	Reach Ring	47	4.81	93	5.33	0.54	30
2	Grasp & Lift Ring	39	4.50	80	4.87	0.56	25
3	Transfer / Move to Peg	51	4.99	111	5.59	0.53	29
4	Place Ring on Peg	42	4.50	91	5.05	0.59	26
5	Return / Release	14	3.73	13	3.64	0.16	14

In summary, SPARTAN presents *higher per-phase action entropy*, *lower action concentration*, and *richer target diversity* than CholecT50, despite being a standardised bench-top task. Combined with the bimanual coordination requirement, these properties position SPARTAN as a controlled yet non-trivial benchmark that isolates action-reasoning challenges from the visual noise and class-imbalance issues of in-vivo surgical data.

References

[1] C.I. Nwoye *et al.*, "Rendezvous: Attention mechanisms for the recognition of surgical action triplets in endoscopic videos", *Medical Image Analysis*, vol. 78, 2022.

[2] Kevin P. Murphy, "Machine learning: a probabilistic perspective", *MIT press*, 2012.
